# Continuous-Flow Synthesis of Deuterium-Labeled Antidiabetic Chalcones: Studies towards the Selective Deuteration of the Alkynone Core

**DOI:** 10.3390/molecules21030318

**Published:** 2016-03-07

**Authors:** Sándor B. Ötvös, Chi-Ting Hsieh, Yang-Chang Wu, Jih-Heng Li, Fang-Rong Chang, Ferenc Fülöp

**Affiliations:** 1 Institute of Pharmaceutical Chemistry, University of Szeged, Eötvös u. 6, H-6720 Szeged, Hungary; otvossandor@pharm.u-szeged.hu (S.B.Ö.); u98831001@gmail.com (C.-T.H.); 2 MTA-SZTE Stereochemistry Research Group, Hungarian Academy of Sciences, Eötvös u. 6, H-6720 Szeged, Hungary; 3 Graduate Institute of Natural Products, College of Pharmacy, Kaohsiung Medical University, Kaohsiung 807, Taiwan; 4 School of Pharmacy, College of Pharmacy, China Medical University, Taichung 40402, Taiwan; yachwu@mail.cmu.edu.tw; 5 Chinese Medicine Research and Development Center, China Medical University Hospital, Taichung 40447, Taiwan; 6 Ph.D. Program in Toxicology and School of Pharmacy, Kaohsiung Medical University, Kaohsiung 807, Taiwan; jhlitox@kmu.edu.tw; 7 Center for Infectious Disease and Cancer Research, Kaohsiung Medical University, Kaohsiung 807, Taiwan

**Keywords:** chalcones, continuous-flow reactor, deuterium labeling, heterogeneous catalysis, triple bond reduction

## Abstract

Flow chemistry-based syntheses of deuterium-labeled analogs of important antidiabetic chalcones were achieved via highly controlled partial C≡C bond deuteration of the corresponding 1,3-diphenylalkynones. The benefits of a scalable continuous process in combination with on-demand electrolytic D_2_ gas generation were exploited to suppress undesired over-reactions and to maximize reaction rates simultaneously. The novel deuterium-containing chalcone derivatives may have interesting biological effects and improved metabolic properties as compared with the parent compounds.

## 1. Introduction

**Figure 1:**
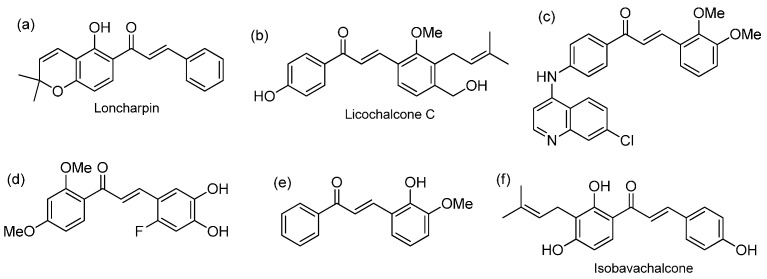
Examples of chalcone derivatives with various biological effects: (**a**) antiplatelet activity [8]; (**b**) antibacterial activity [6]; (**c**) antitubercular activity [9]; (**d**) anti-inflammatory effect [7]; (**e**) antiviral activity [5]; (**f**) anticancer activity [4].

Chalcones are 1,3-diaryl-2-propen-1-ones which belong to the flavonoid family [1,2,3]. Their naturally occurring representatives and synthetic analogs exert a wide array of pharmacological activities, including anticancer [4], antiviral [5], antibacterial [6], anti-inflammatory [7], antiplatelet [8] and antitubercular [9] effects (Figure 1), making the chalcone skeleton an important scaffold for drug discovery [10,11,12,13,14].

**Figure 2:**
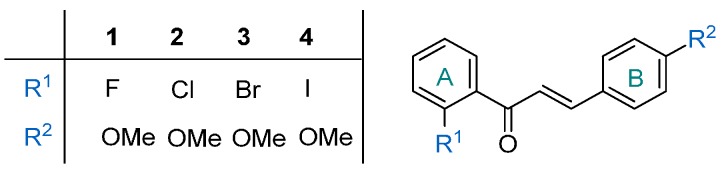
Antidiabetic chalcone derivatives (**1**–**4**) developed by our research group.

In recent decades, the metabolic syndrome and diabetes have become ever-increasing health issues worldwide [15]. The associated complications of these disorders, such as stroke, cardiovascular diseases, peripheral vascular diseases, diabetic neuropathy, renal failure, blindness and amputations, lead to reduced life expectancy, the enhancement of disability, and enormous medical costs [16,17,18]. Despite extensive biological studies on chalcones [10,11,12,13,14], their antidiabetic activities have scarcely been investigated [19,20,21]. Our research group has previously reported that certain synthetic chalcones (**1**‒**4**) containing various halogen atoms at position 2 of ring A (Figure 2) effectively promote glucose consumption in adipocytes [22]. Structure–activity relationship analyses have established that, besides the halogen substituents on ring A, the presence of a methoxy group on ring B contributes to the potent antidiabetic activities observed.

There has recently been an upsurge of interest in isotopic labeling studies not merely in chemistry and biochemistry [23,24,25,26], but also in environmental sciences and pharmacological research [27,28]. Among the stable isotopes used for labeling studies, deuterium is of marked significance [24,25,26,27], thanks to the fact that it has the highest relative mass difference compared to its light isotope (^1^H), which allows a considerably large isotope effect [29]. Deuterium not only serves as a nonradioactive, stable isotopic tracer in the evaluation of the metabolic pathways [30] or in tracer studies to investigate pharmacokinetics and reaction mechanisms [31,32]; as a result of the large isotope effect, H‒D exchange may also potentiate active pharmaceutical ingredients [27]. As an example, the metabolic rates of nonlabeled derivatives and deuterated compounds are very different, and the use of deuterium-labeled analogs may therefore help in the elimination of unwanted metabolites, which can lead in practice to the diminution of adverse effects [33,34,35].

**Scheme 1:**
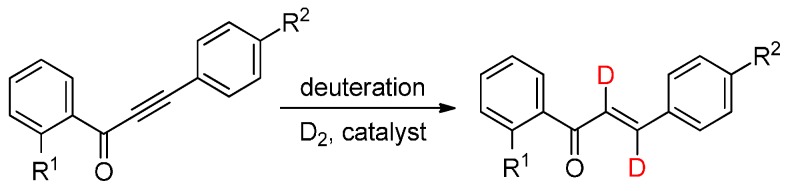
The proposed synthesis of deuterium-labeled chalcones.

Inspired by the above findings, we aimed for the synthesis of deuterium-labeled derivatives of antidiabetic chalcones **1**‒**4** by exploring the partial deuteration of the C≡C bond of the corresponding 1,3-diphenylalkynone analogs (Scheme 1). We presumed that the incorporation of deuterium onto the α,β-unsaturated carbonyl system may not only help potentiate the pharmacological activities, but also exert positive effects on the metabolic pathway of the bioactive chalcones [27]. Selective deuteration of the alkynone core may be expected to constitute a significant synthetic challenge in view of the easy over-reduction of the C=C bond and the carbonyl group [36]; we therefore set out to exploit the benefits of continuous-flow processing [37,38,39,40,41,42,43,44,45,46,47].

## 2. Results and Discussion

Most techniques for the synthesis of deuterium-labeled compounds rely on expensive D_2_ gas as a deuterium source. Similarly to hydrogenations, the handling of D_2_ gas demands extreme precautions and costly special apparatus to maintain sufficient operational safety. The conventional methods for the production of D_2_ gas are fractional distillation of liquid hydrogen, pyrolysis of UD_3_, and reactions of D_2_O with reducing agents (Na, Fe or Mg) [48]. Unfortunately, these methods involve several drawbacks, such as the production of radioactive waste, the need for cryogenic conditions, and the large quantity of metal sludge produced [48]. Catalytic H–D exchange reactions between H_2_ and D_2_O appear more feasible, but often do not yield sufficiently pure D_2_ and require long reaction times [49,50,51,52,53,54]. Alternatively, the application of deuterated reagents and deuterated solvents as isotopic sources eliminates the hazards of gas handling, but the costs of these special conditions severely limit the practical applicability [55,56,57,58,59].

**Figure 3:**
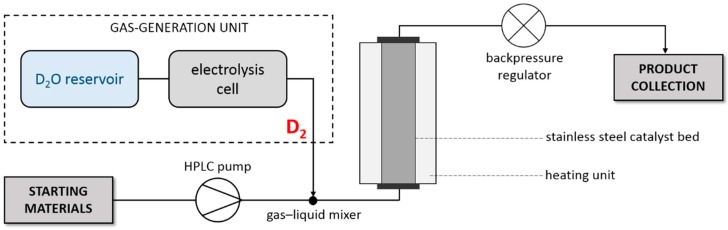
Schematic outline of the continuous-flow deuteration reactor (H-Cube^®^).

To overcome the difficulties of conventional deuterium labeling techniques, we have developed a unique flow chemistry-based method for deuteration reactions by changing the hydrogen source to deuterated water in an H-Cube^®^ system (Figure 3) [36,60,61]. This approach is safe, as D_2_ gas is continuously generated in small aliquots through the electrolytic decomposition of D_2_O, the consumption of which is very low, implying high deuterium efficiency. The gas generated *in situ* is combined with the substrate solution via a gas–liquid mixer, and the resulting mixture is transported to a filled catalyst bed, where the triphasic reaction takes place [62,63]. The purity of the D_2_ gas produced can be as high as 99.9%, which ensures high deuterium incorporation ratios [36,60,61]; moreover, the precise control over the reaction conditions may enhance the efficacy of the deuteration reactions [36,39,47,64,65].

**Scheme 2:**
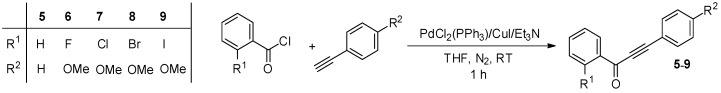
Coupling of acid chlorides with terminal acetylenes under Sonogashira conditions to yield the corresponding ynones as starting materials for the continuous-flow deuterations.

Our synthetic strategy toward the anticipated deuterium-labeled compounds involved continuous-flow deuteration of the corresponding ynone analogs of antiabetic chalcones **1**–**4** (Scheme 1). Ynones **5**–**9** were prepared by literature procedures through the coupling of acid chlorides with terminal acetylenes under Sonogashira conditions: PdCl_2_(PPh_3_)_2_/CuI as a catalyst and Et_3_N as a base in THF as solvent (Scheme 2) [66,67,68]. The mixtures were stirred for 1 h at room temperature (RT) under a N_2_ atmosphere, and ynones **5**–**9** were achieved in excellent yields after simple extractive work-up and chromatographic purification (see Experimental Section).

We first investigated the deuteration of 1,3-diphenylprop-2-yn-1-one (**5**) as a nonsubstituted model compound (Table 1). The reactions were carried out in EtOAc as aprotic solvent so as to prevent D–H exchange and to maximize deuterium incorporation. We had previously established that the steric hindrance generated by the halogen atom at position 2 of ring A exerts a significant influence on the rate of deuterium incorporation, as larger substituents may block the central core of the molecule, necessitating harsher conditions for the reaction to take place [36]. The deuteration of **5** was therefore investigated by using Lindlar catalyst (5% Pd on CaCO_3_, poisoned with lead) at ambient temperature (25 °C) and at pressures in the range 10‒80-bar. The utilization of high pressures in gas–liquid–solid reactions is favorable, as the elevation of pressure increases the solubility of gases, thereby enhancing the reaction rate. Accordingly, it was found that pressurization pushed the reaction to completion, resulting in higher total conversions, though pressures ≥40 bar contributed significantly to over-reaction of the desired dideuteroenone (**5a**) to give the corresponding tetradeuteroketone (**5b**) and pentadeuteroalcohol (**5c**) as side products (Table 1). At 80 bar, for example, a total conversion of 82% was found, but the crude product mixture contained 23% of **5b**, and even **5c** was formed to an extent of 4% (entry 4). If it is taken into account that **5a** can be isolated from the alkynone starting material (**5**) more easily than from the over-reaction products (**5b** and **5c**), 20 bar can be selected as the pressure of choice, with an acceptable total conversion of 57% and an excellent dideuteroenone/tetradeuteroketone ratio of 86:14, and without detectable pentadeuteroalcohol formation (entry 2). During the reactions, the (*Z*) deuterated chalcone isomer is formed, as a consequence of the mechanism of the heterogeneous catalytic process [69]. However, the (*Z*) chalcone readily undergoes thermal or photochemical isomerization to the more stable (*E*) form [70,71,72], which was detected exclusively after the purification. It must also be noted that an O–D bond on the pentadeuterochalcone side-product (**5c**) was not observed, as it is converted instantly to O–H due to exchange with moisture when it is exposed to air.

In continuous-flow deuteration of 1-(2-fluorophenyl)-3-(4-methoxyphenyl)prop-2-yn-1-one (**6**) with Lindlar catalyst, the gradual increase of the pressure from 10 to 80 bar at ambient temperature had a pronounced influence on the rate of the reaction (Table 2). Although lower overall conversions were achieved than in deuterations of the nonsubstituted alkynone (**5**), compound **6** exhibited a lower tendency to over-reaction, even at pressures ≥40 bar, which could possibly be explained by the steric hindrance generated by the fluorine atom at position 2 of ring A. Thus, 80 bar, selected as optimum pressure, resulted in a total conversion of 63% and a dideuteroenone/tetradeuteroketone ratio of 80:20 without over-reaction to **6c** (entry 4).

When the fluorine atom on phenyl ring A was replaced by chlorine (1-(2-chlorophenyl)-3-(4-methoxyphenyl)prop-2-yn-1-one, **7**), deuteration with Lindlar catalyst at 20‒80 bar resulted in conversions of only 33%‒47% (Table 3, entries 1‒3). 5% Pd/BaSO_4_ was therefore investigated next as a more active heterogeneous catalyst (keeping in mind that activated charcoal as a catalyst support may contain protic contamination that can negatively influence deuterium incorporation) [73]. The Pd/BaSO_4_-mediated reactions at RT gave excellent total conversions (75% and 86% at 20 and 40 bar, respectively), but formation of the desired dideuteroenone **7a** was significantly suppressed by undesired over-reduction to **7b**, and even to **7c**, affording dideuteroenone/tetradeuteroketone/pentadeuteroalcohol ratios of 62:38:0 and 60:38:2, at 20 and 40 bar, respectively (entries 4 and 5).

In the case of bromine-substituted ynone **8** (1-(2-bromophenyl)-3-(4-methoxyphenyl)prop-2-yn-1-one), the efficacy of 5% Pd/BaSO_4_ was tested first at 40 bar employing temperatures of 50 and 70 °C, to compensate the larger steric hindrance generated by the halogen atom at position 2 of ring A (Table 4). Despite the achievement of acceptable conversions of 50% and 53%, significant over-reduction occurred at both temperatures, and dideuteroenone **8a** was formed to extents of only 69% and 56% at 50 and 70 °C, respectively (entries 1 and 2). We were delighted to find that elevation of the pressure to 60 bar at ambient temperature resulted in a slight increase in conversion to 66% and a significant improvement in chemoselectivity, with an acceptable dideuteroenone (**8a**)/tetradeuteroketone (**8b**) ratio of 77:23, without detectable pentadeuteroalcohol (**8c**) present (entry 3). An effort to further enhance the amount of **8a** by raising the temperature to 50 °C was unsuccessful because of the intensified **8a**‒**8b** over-reduction (entry 4).

Finally, the continuous-flow deuteration of 1-(2-iodophenyl)-3-(4-methoxyphenyl)prop-2-yn-1-one (**9**) was attempted by using 5% Pd/BaSO_4_ as catalyst in combination with harsher reaction conditions, with regard to the significant steric hindrance generated by iodine (Table 5) [36]. Although the total conversion at 80 bar and 50 °C was only 17%, formation of the corresponding dideuteroenone (**9a**) was exclusive under these conditions (entry 1). We were pleased to find that when the temperature was elevated to 100 °C, the conversion rose to an acceptable level of 56%, still without the formation of over-reaction products (entry 2). In attempts to improve the rate of the reaction, the use of 5% Pt/Al_2_O_3_ as a more active heterogeneous catalyst led to higher total conversions at RT and 20‒80 bar, but it had a pronounced negative effect on the chemoselectivity, with the unwanted tetradeuteroketone (**9b**) becoming the main product, together with significant amounts of pentadeuteroalcohol **9c** (entries 3‒5).

The above optimization reactions (Table 1, Table 2, Table 3, Table 4 and Table 5) were typically carried out on a 0.1 mmol scale. For preparative purposes, a 10-fold scale-up was made under the selected conditions (see Table 1, entry 2; Table 2, entry 4; Table 3, entry 4; Table 4, entry 3; and Table 5, entry 2) simply by pumping in a larger amount of starting material. Even gram-scale deuterations can readily be performed in flow as a function of the starting material quantity and the operation time, without any change in the reaction conditions and without the need for re-optimization.

As a consequence of the high purity of the D_2_ gas employed, the deuterated target compounds were obtained with deuterium contents (which reflect the deuterium incorporation ratio over incidental hydrogen addition) of ≥98%. It should also be noted that neither over-reaction products containing reduced or partially reduced aromatic rings, nor halogen–deuterium exchange on ring A were detected, even when Pt/Al_2_O_3_ was used as a catalyst.

## 3. Materials and Methods

### 3.1. Materials

The reagents and materials were of the highest commercially available grade and were used without further purification. Cartridges containing 5% Pt/Al_2_O_3_, 5% Pd/BaSO_4_ and Lindlar catalyst were purchased from ThalesNano Inc. (Budapest, Hungary).

### 3.2. Synthesis of the Starting Materials

The corresponding acid chloride (1.0 mmol, 1 equiv.), terminal acetylene (1.0 equiv.), PdCl_2_(PPh_3_)_2_ (0.03 equiv.), CuI (0.06 equiv.) and Et_3_N (1.5 equiv.) were combined in THF (6 mL), and the mixture was stirred for 1 h at ambient temperature under a N_2_ atmosphere. It was next diluted with water to 12 mL, and the resulting solution was extracted with CH_2_Cl_2_ (2 × 15 mL). The combined organic layers were dried over Na_2_SO_4_ and concentrated under reduced pressure. The residue obtained was purified by column chromatography on silica gel, with a mixture of *n*-hexane/EtOAc as eluent; ynones **5**‒**9** were achieved in yields of 83%‒92% [66,67,68].

### 3.3. Continuous-Flow Deuterations

Deuterations were carried out in an H-Cube^®^ system (ThalesNano Inc.) with replacement of the H_2_O hydrogen source to D_2_O (VWR, 99.96%) [36,60,61]. The catalyst cartridges, with internal dimensions of 30 × 4 mm, contained approximately 100 mg of 5% Pt/Al_2_O_3_, 5% Pd/BaSO_4_ or Lindlar catalyst. The selected cartridge was placed into a heating unit controlled by a built-in Peltier system (maximum temperature: 100 °C), and the system also contained a backpressure regulator that ensured constant pressures up to a maximum of 100 bar. The continuous stream of the reaction solution was provided by an HPLC pump (Knauer WellChrom K-120). For each reaction, a 1-mg·mL^−1^ solution of the corresponding ynone (**5**‒**9**) was prepared in ethyl acetate (HPLC grade). The mixture was homogenized by sonication for 2 min and then pumped through the H-Cube^®^ under the appropriate conditions. Between two reactions, the catalyst bed was washed for 10 min with ethyl acetate at 1 mL min^‒1^. The crude products were purified by column chromatography on silica gel with mixtures of *n*-hexane/EtOAc as eluent.

### 3.4. Product Analysis

Ynones **5**‒**9** and deuterium-labeled chalcones **5a**‒**9a** were characterized by NMR and GC-MS. ^1^H-NMR and ^13^C-NMR spectra were recorded in CDCl_3_ as an applied solvent on Bruker Avance DRX 400 and JEOL ECS 400 instruments with TMS as the internal standard, at 400.1 and 100.6 MHz, respectively. GC-MS analyses were carried out with a Thermofisher Scientific DSQ II Single Quadrupole GC/MS, on a 30 m × 0.25 mm × 0.25 μm HP-5MS capillary column (Agilent J & W Scientific). The measurement parameters: column oven temperature: from 50 to 300 °C at 10 °C·min^−1^ (0‒25 min), and 300 °C (25‒30 min); injection temperature: 250 °C; ion source temperature: 200 °C; EI: 70 eV; carrier gas: He, at 1 mL·min^‒1^; injection volume: 5 μL; split ratio: 1:50; and mass range: 45–800 *m*/*z*. Conversions and product ratios were determined from the GC-MS spectra of the crude materials. Deuterium contents were calculated from the relative intensities of the ^1^H-NMR indicator signals. Product characterization data can be found in the Supporting Information.

## 4. Conclusions

Scalable continuous-flow syntheses of the deuterium-labeled derivatives of antidiabetic chalcones were achieved in the present study. Our synthetic strategy relied on the selective C≡C bond deuteration of the corresponding alkynone analogs in an H-Cube^®^ system following change of the hydrogen source to high-purity deuterated water. The methodology applied was simple and safe as it eliminated potentially dangerous D_2_ gas-handling. The effects of the most important reaction conditions (catalyst, pressure and temperature) were investigated in order to determine the optimum deuteration conditions. In spite of the possibility of facile over-reactions, the desired deuterium-labeled compounds were attained with high selectivities.

A patent application has been filed on deuterium-labeled chalcones **6a**‒**9a**. Their biological effects and kinetic profiles are currently under investigation in our laboratories; these results will be reported in due course.

## Figures and Tables

**Table 1:** Continuous-flow deuteration of **5** ^a^. 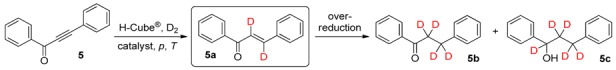

Entry	Catalyst	*p* (bar)	*T* (°C)	Total Conversion ^b^ (%)	Product Ratio ^b^ (%)		
					5a ^c^	5b	5c
1	Lindlar catalyst	10	25	47	92	8	0
2	Lindlar catalyst	20	25	57	86	14	0
3	Lindlar catalyst	40	25	63	77	23	0
4	Lindlar catalyst	80	25	82	73	23	4

^a^: Conditions: *c* = 1 mg·mL^‒1^ in ethyl acetate, 1 mL·min^–1^ flow rate; ^b^: Determined by GC-MS analysis of the crude material; ^c^: During the reactions, the (*Z*) deuterated chalcone isomer is formed, but readily isomerizes to the more stable (*E*) form.

**Table 2:** Continuous-flow deuteration of **6** ^a^. 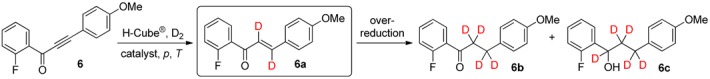

Entry	Catalyst	*p* (bar)	*T* (°C)	Total Conversion ^b^ (%)	Product Ratio ^b^ (%)		
					6a ^c^	6b	6c
1	Lindlar catalyst	10	25	23	90	10	0
2	Lindlar catalyst	20	25	40	86	14	0
3	Lindlar catalyst	40	25	57	81	19	0
4	Lindlar catalyst	80	25	63	80	20	0

^a^: Conditions: *c* = 1 mg·mL^‒1^ in ethyl acetate, 1 mL·min^–1^ flow rate; ^b^: Determined by GC-MS analysis of the crude material; ^c^: During the reactions, the (*Z*) deuterated chalcone isomer is formed, but readily isomerizes to the more stable (*E*) form.

**Table 3:** Continuous-flow deuteration of **7** ^a^. 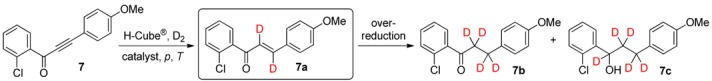

Entry	Catalyst	*p* (bar)	*T* (°C)	Total Conversion ^b^ (%)	Product Ratio ^b^ (%)		
					7a ^c^	7b	7c
1	Lindlar catalyst	20	25	33	89	11	0
2	Lindlar catalyst	40	25	43	88	12	0
3	Lindlar catalyst	80	25	47	79	21	0
4	5% Pd/BaSO_4_	20	25	75	62	38	0
5	5% Pd/BaSO_4_	40	25	86	60	38	2

^a^: Conditions: *c* = 1 mg·mL^‒1^ in ethyl acetate, 1 mL·min^–1^ flow rate; ^b^: Determined by GC-MS analysis of the crude material; ^c^: During the reactions, the (*Z*) deuterated chalcone isomer is formed, but readily isomerizes to the more stable (*E*) form.

**Table 4:** Continuous-flow deuteration of **8** ^a^. 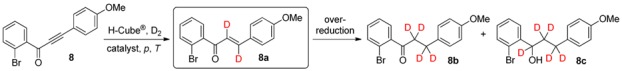

Entry	Catalyst	*p* (bar)	*T* (°C)	Total Conversion ^b^ (%)	Product Ratio ^b^ (%)		
					8a ^c^	8b	8c
1	5% Pd/BaSO_4_	40	50	50	69	31	0
2	5% Pd/BaSO_4_	40	70	53	56	44	0
3	5% Pd/BaSO_4_	60	25	66	77	23	0
4	5% Pd/BaSO_4_	60	50	82	61	39	0

^a^: Conditions: *c* = 1 mg·mL^‒1^ in ethyl acetate, 1 mL·min^–1^ flow rate; ^b^: Determined by GC-MS analysis of the crude material; ^c^: During the reactions, the (*Z*) deuterated chalcone isomer is formed, but readily isomerizes to the more stable (*E*) form.

**Table 5:** Continuous-flow deuteration of **9** ^a^. 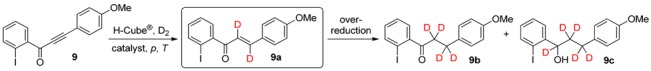

Entry	Catalyst	*p* (bar)	*T* (°C)	Total Conversion ^b^ (%)	Product Ratio ^b^ (%)		
					9a ^c^	9b	9c
1	5% Pd/BaSO_4_	80	50	17	100	0	0
2	5% Pd/BaSO_4_	80	100	56	100	0	0
3	5% Pt/Al_2_O_3_	20	25	89	28	67	5
4	5% Pt/Al_2_O_3_	40	25	95	18	68	14
5	5% Pt/Al_2_O_3_	80	25	99	7	59	34

^a^: Conditions: *c* = 1 mg·mL^‒1^ in ethyl acetate, 1 mL·min^–1^ flow rate; ^b^: Determined by GC-MS analysis of the crude material; ^c^: During the reactions, the (*Z*) deuterated chalcone isomer is formed, but readily isomerizes to the more stable (*E*) form.

## Supplementary Materials

Supplementary materials can be accessed at: http://www.mdpi.com/1420-3049/21/3/318/s1.
