# Supplementary material for: Continuous-Flow Synthesis of Deuterium-Labeled Antidiabetic Chalcones: Studies towards the Selective Deuteration of the Alkynone Core
Source: Molecules. 2016 Mar 7;21(3):318. doi: 10.3390/molecules21030318 (PMC6273355; doi:10.3390/molecules21030318)
Supplement: Supplementary file 1 [file molecules-21-00318-s001.pdf]

Supplementary Materials: Continuous-Flow Synthesis of Deuterium-Labeled Antidiabetic Chalcones: Studies towards the Selective Deuteration of the Alkynone Core

Sándor B. Ötvös, Chi-Ting Hsieh, Yang-Chang Wu, Jih-Heng Li, Fang-Rong Chang and Ferenc Fülöp

Table of Contents

1. Analytical data S2

2. Collection of NMR spectra S5

1,3-diphenylprop-2-yn-1-one (**5**)

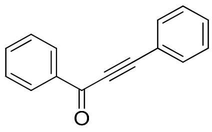

NMR data is in agreement with the literature [1]. ^{1}H-NMR (400.1 MHz, CHLOROFORM-*d*) δ ppm 7.20–7.45 (m, 5H), 7.45–7.61 (m, 3H), 8.09–8.23 (m, 2H); 8.15 (d, *J* = 7.86 Hz, 2H); ^{13}C-NMR (100.6 MHz, CHLOROFORM-*d*) δ ppm 87.5 (1C), 93.9 (1C), 120.30 (1C), 129.08 (2C), 129.13 (2C), 129.87 (2C), 131.25 (1C), 133.42 (2C), 134.53 (1C), 137.01 (1C), 178.25 (1C); MS (EI): *m*/*z* (%): 205.97, 178.11, 129.13.

1-(2-Fluorophenyl)-3-(4-methoxyphenyl)prop-2-yn-1-one (**6**)

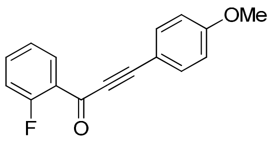

^{1}H-NMR (400 MHz, CHLOROFORM-*d*) δ ppm 3.89 (s, 3H), 7.00 (d, *J* = 8.56 Hz, 2H), 7.23–7.32 (m, 1H), 7.38 (t, *J* = 7.55 Hz, 1H), 7.67 (d, *J* = 8.31 Hz, 3H), 8.21 (t, *J* = 7.55 Hz, 1H); ^{13}C-NMR (101 MHz, CHLOROFORM-*d*) δ ppm 54.69 (1C) 87.24 (1C), 93.16 (1C), 111.27 (1C), 113.90 (2C), 115.57–117.45 (1C), 123.68 (1C), 131.15 (1C) ,134.63 (2C), 134.87 (1C), 159.41 (1C), 160.75 (1C), 162.27 (1C), 172.22 (1C); MS (EI): *m*/*z* (%): 254.11, 226.10, 211.08, 183.13, 159.14; elemental analysis calcd. (%) for $C_{16}H_{11}FO_2$: C 75.58; H 4.36; found: C 75.61; H 4.33.

1-(2-Chlorophenyl)-3-(4-methoxyphenyl)prop-2-yn-1-one (**7**)

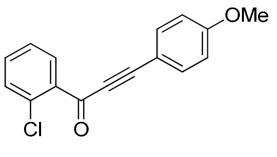

NMR data is in agreement with the literature [2]. ^{1}H-NMR (400.1 MHz, CHLOROFORM-*d*) δ ppm 3.74 (s, 3H), 6.83 (d, *J* = 8.81 Hz, 2H), 7.29–7.41 (m, 3H), 7.50 (d, *J* = 8.56 Hz, 2H), 8.01 (d, *J* = 7.55 Hz, 1H); ^{13}C-NMR (100.6 MHz, CHLOROFORM-*d*) δ ppm 55.40 (1C), 88.49 (1C), 95.31 (1C), 111.55

(1C), 114.49 (2C), 126.85 (1C), 131.43 (1C), 132.40 (1C), 133.24 (1C), 134.71 (1C), 135.14 (2C), 135.96 (1C), 161.94 (1C), 176.55 (1C); MS (EI): *m*/*z* (%): 270.02, 242.09, 227.07, 159.15.

1-(2-Bromophenyl)-3-(4-methoxyphenyl)prop-2-yn-1-one (**8**)

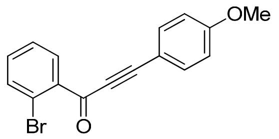

NMR data is in agreement with the literature [3]. ^{1}H-NMR (400.1 MHz, CHLOROFORM-*d*) δ ppm 3.82 (s, 3H), 6.90 (d, *J* = 8.06 Hz, 2H), 7.34 (t, *J* = 8.06 Hz, 1H), 7.43 (t, *J* = 7.81 Hz, 1H), 7.57 (d, *J* = 7.55 Hz, 2H), 7.66 (d, *J* = 7.81 Hz, 1H), 8.02 (d, *J* = 7.81 Hz, 1H); ^{13}C-NMR (100.6 MHz, CHLOROFORM-*d*) δ ppm 55.20 (1C), 87.93 (1C), 95.34 (1C), 111.17 (1C), 114.32 (2C), 120.71 (1C), 127.28 (1C), 132.44 (1C), 133.06 (1C), 134.61 (1C), 134.93 (2C), 137.28 (1C), 161.72 (1C), 176.87 (1C); MS (EI): *m*/*z* (%): 314.08, 288.14, 273.22, 159.34.

1-(2-Iodophenyl)-3-(4-methoxyphenyl)prop-2-yn-1-one (**9**)

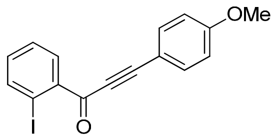

NMR data is in agreement with the literature [4]. ^{1}H-NMR (400.1 MHz, CHLOROFORM-*d*) δ ppm 3.75 (s, 3H), 6.84 (d, *J* = 8.81 Hz, 2H), 7.08–7.15 (m, 1H), 7.44 (t, *J* = 7.55 Hz, 1H), 7.51 (d, *J* = 8.81 Hz, 2H), 7.95 (d, *J* = 8.06 Hz, 1H), 8.05 (d, *J* = 7.81 Hz, 1H); ^{13}C-NMR (100.6 MHz, CHLOROFORM-*d*) δ ppm 55.18 (1C), 87.05 (1C), 92.41 (1C), 95.48 (1C), 111.12 (1C), 114.19 (2C), 127.81 (1C), 132.48 (1C), 132.98 (1C), 134.84 (2C), 137.92 (1C), 141.64 (1C), 161.58 (1C), 177.56 (1C); MS (EI): *m*/*z* (%): 361.96, 281.09, 207.10.

(E)-2,3-Dideutero-1,3-diphenylprop-2-en-1-one (**5a**)

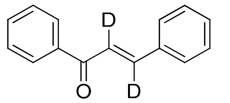

^{1}H-NMR (400.1 MHz, CHLOROFORM-*d*) δ ppm 7.38–7.48 (m, 3H), 7.48–7.56 (m, 2H), 7.57–7.63 (m, 1H), 7.63–7.72 (m, 2H), 8.04 (d, *J* = 7.79 Hz, 2H); ^{13}C-NMR (100.6 MHz, CHLOROFORM-*d*) δ ppm 121.51 (1C), 128.36 (2C), 128.41 (2C), 128.55 (2C), 128.87 (2C), 130.48 (1C), 132.72 (1C), 134.66 (1C), 138.08 (1C), 143.77 (1C), 190.41 (1C); MS (EI): *m*/*z* (%): 209.19, 181.26, 105.17; elemental analysis calcd. (%) for $C_{15}H_{10}D_{2}O$: C 85.68; H 6.71; found: C 85.72; H 6.68.

(E)-2,3-Dideutero-1-(2-fluorophenyl)-3-(4-methoxyphenyl)prop-2-en-1-one (**6a**)

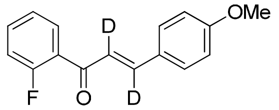

^{1}H-NMR (400.1 MHz, CHLOROFORM-*d*) δ ppm 3.83 (s, 3H), 6.91 (d, *J* = 8.24 Hz, 2H), 7.14 (dd, *J* = 9.85, 8.93 Hz, 1H), 7.23 (t, *J* = 7.56 Hz, 1H), 7.45–7.52 (m, 1H), 7.56 (d, *J* = 8.70 Hz, 2H), 7.79 (t, *J* = 7.33 Hz, 1H); ^{13}C-NMR (100.6 MHz, CHLOROFORM-*d*) δ ppm 55.35 (1C), 114.36 (2C), 116.10 (1C), 123.18 (1C), 124.40 (1C), 127.23 (1C), 127.39 (1C), 130.37 (2C), 130.85 (1C), 133.59 (1C), 144.38 (1C),

159.77 (1C), 161.48 (1C), 189.03 (1C); MS (EI): *m*/*z* (%):258.05, 243.12, 227.14, 163.14; elemental analysis calcd. (%) for $C_{16}H_{11}D_2FO_2$: C 74.40; H 5.85; found: C 74.45; H 5.81.

(E)-2,3-Dideutero-1-(2-chlorophenyl)-3-(4-methoxyphenyl)prop-2-en-1-one (**7a**)

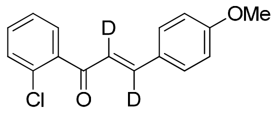

^{1}H-NMR (400.1 MHz, CHLOROFORM-*d*) δ ppm 3.85 (s, 3H), 6.92 (d, *J* = 8.70 Hz, 2H), 7.33–7.38 (m, 1H), 7.41 (td, *J* = 7.67, 1.60 Hz, 1H), 7.44–7.49 (m, 2H), 7.52 (d, *J* = 8.70 Hz, 2H); ^{13}C-NMR (100.6 MHz, CHLOROFORM-*d*) δ ppm 55.39 (1C), 114.42 (2C), 123.99 (1C), 126.74 (1C), 126.95 (1C), 129.21 (1C), 130.20 (1C), 130.39 (2C), 131.14 (1C), 131.17 (1C), 139.29 (1C), 144.83 (1C), 161.93 (1C), 193.93 (1C); MS (EI): *m*/*z* (%):274.04, 243.12, 163.14, 135.14; elemental analysis calcd. (%) for $C_{16}H_{11}D_2ClO_2$: C 69.95; H 5.50; found: C 69.98; H 5.47.

(E)-2,3-Dideutero-1-(2-bromophenyl)-3-(4-methoxyphenyl)prop-2-en-1-one (**8a**)

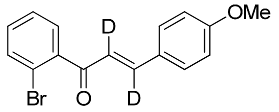

^{1}H-NMR (400.1 MHz, CHLOROFORM-*d*) δ ppm 3.85 (s, 3H), 6.92 (d, *J* = 8.70 Hz, 2H), 7.30–7.37 (m, 1H), 7.39–7.46 (m, 2H), 7.52 (d, *J* = 8.70 Hz, 2H), 7.65 (d, *J* = 7.79 Hz, 1H); ^{13}C-NMR (100.6 MHz, CHLOROFORM-*d*) δ ppm 55.41 (1C), 114.45 (2C), 119.44 (1C), 123.84 (1C), 126.95 (1C), 127.27 (1C), 129.05 (1C), 130.41 (2C), 131.13 (1C), 133.34 (1C), 141.33 (1C), 146.64 (1C), 161.97 (1C), 194.84 (1C); MS (EI): *m*/*z* (%):318.11, 303.07, 287.26, 239.34, 196.15, 163.33; elemental analysis calcd. (%) for $C_{16}H_{11}D_2BrO_2$: C 60.21; H 4.74; found: C 60.26; H 4.70.

(E)-2,3-Dideutero-1-(2-iodophenyl)-3-(4-methoxyphenyl)prop-2-en-1-one (**9a**)

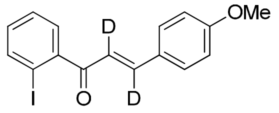

^{1}H-NMR (400.1 MHz, CHLOROFORM-*d*) δ ppm 3.83–3.89 (m, 3H), 6.91–6.96 (m, 2H), 7.17 (td, *J* = 7.67, 1.60 Hz, 1H), 7.33–7.41 (m, 1H), 7.42–7.49 (m, 1H), 7.53 (d, *J* = 8.70 Hz, 2H), 7.94 (d, *J* = 7.79 Hz, 1H); ^{13}C-NMR (100.6 MHz, CHLOROFORM-*d*) δ ppm 55.43 (1C), 92.23 (1C), 114.47 (2C), 123.38 (1C), 127.02 (1C), 127.91 (1C), 128.38 (1C), 130.44 (2C), 131.09 (1C), 139.93 (1C), 144.93 (1C), 147.07 (1C), 161.99 (1C), 196.25 (1C); MS (EI): *m*/*z* (%):364.94, 334.01, 238.14, 195.14, 162.13; elemental analysis calcd. (%) for $C_{16}H_{11}D_2IO_2$: C 52.48; H 4.13; found: C 52.45; H 4.16.

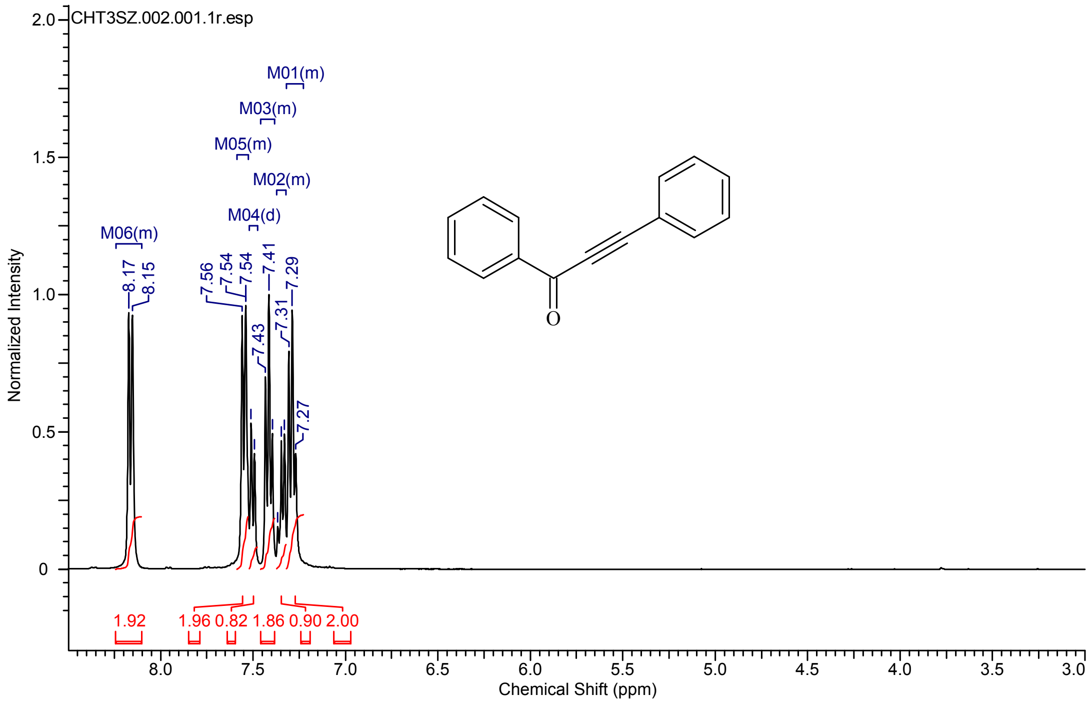

Figure S1. ^{1}H-NMR analysis of compound **5**.

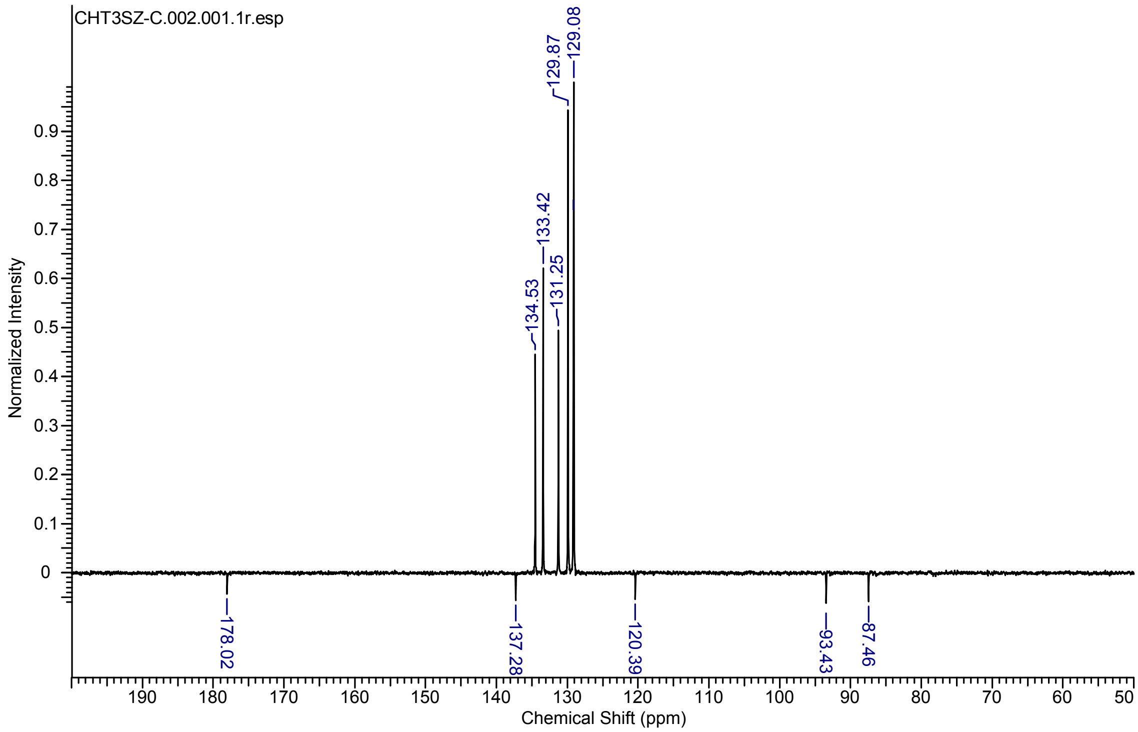

Figure S2. ^{13}C-NMR analysis of compound **5**.

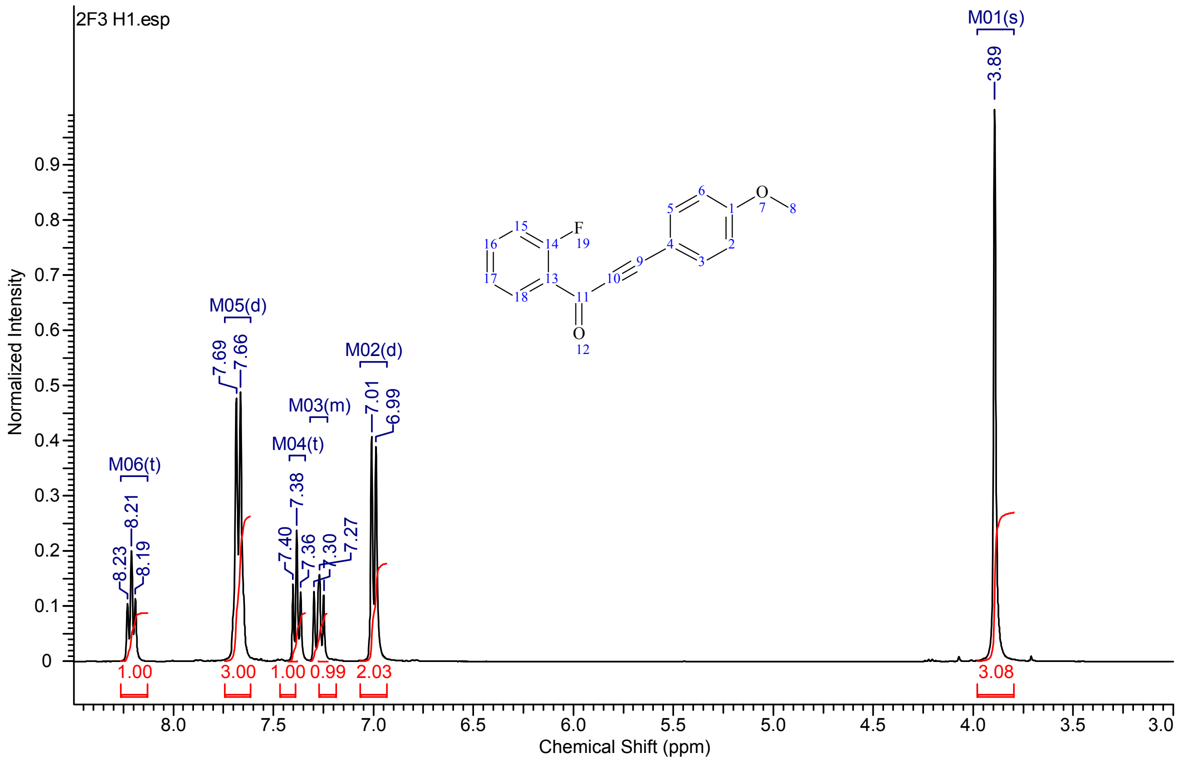

Figure S3. ^{1}H-NMR analysis of compound **6**.

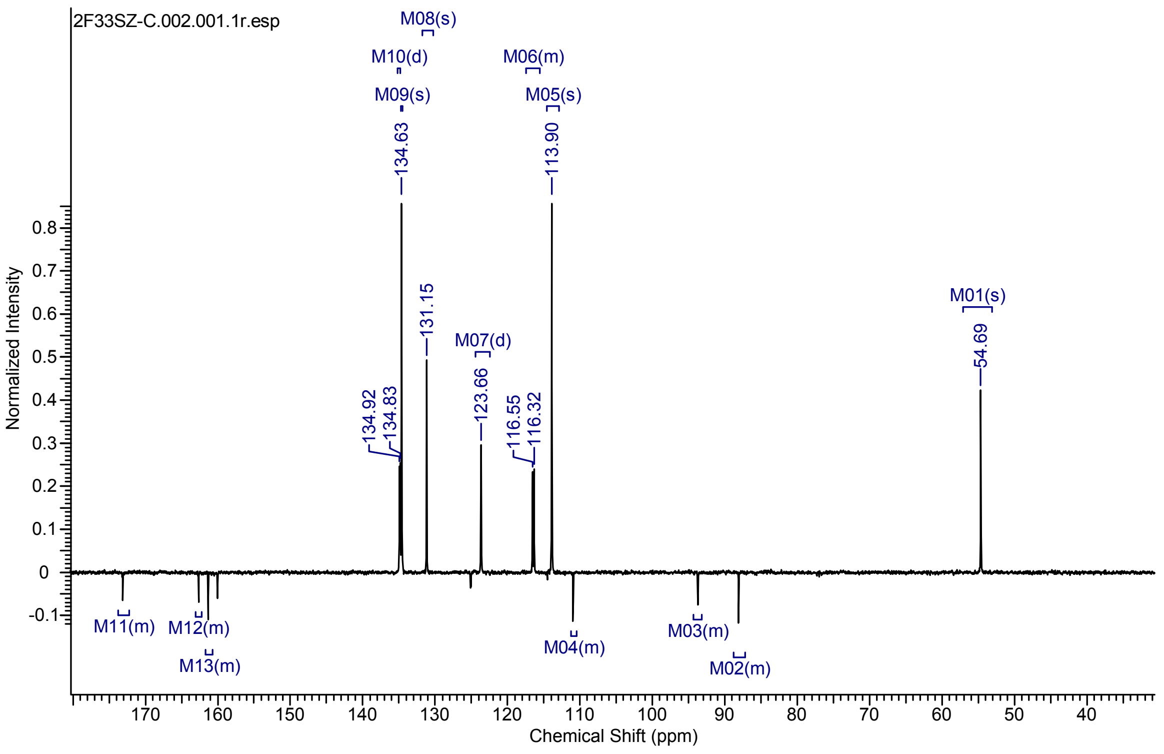

Figure S4. ^{13}C-NMR analysis of compound **6**.

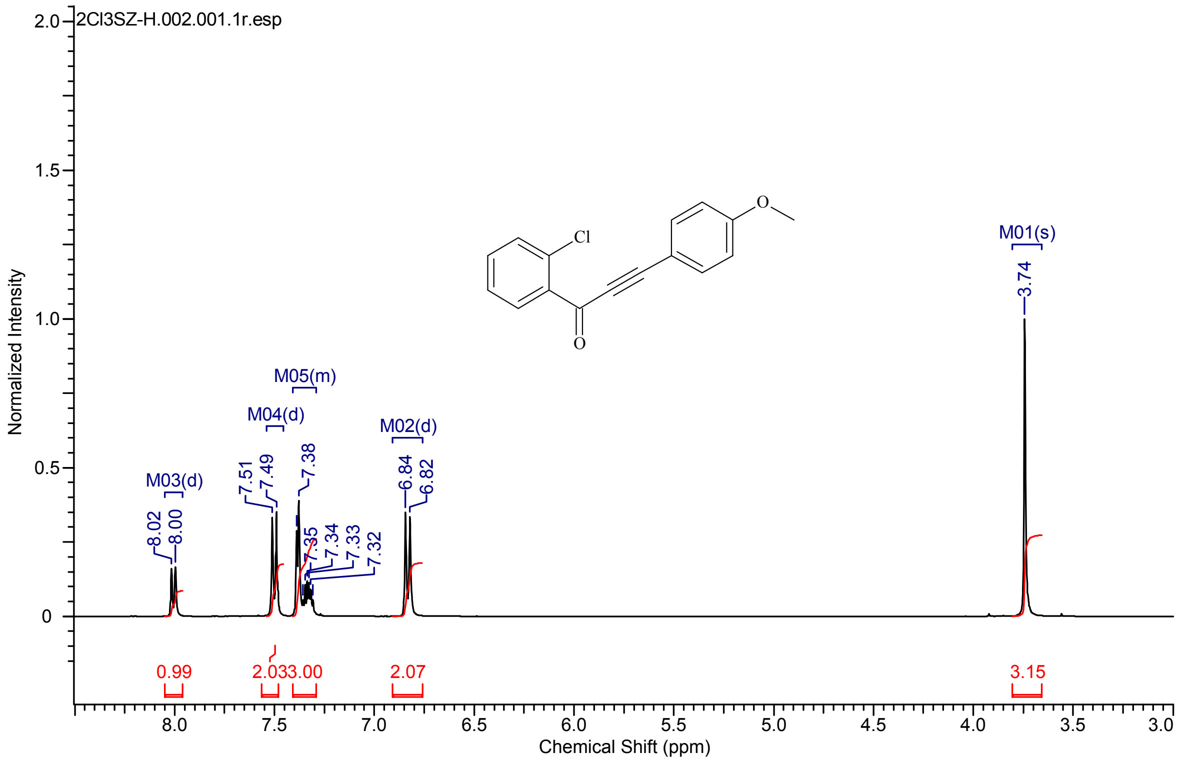

Figure S5. ^{1}H-NMR analysis of compound **7**.

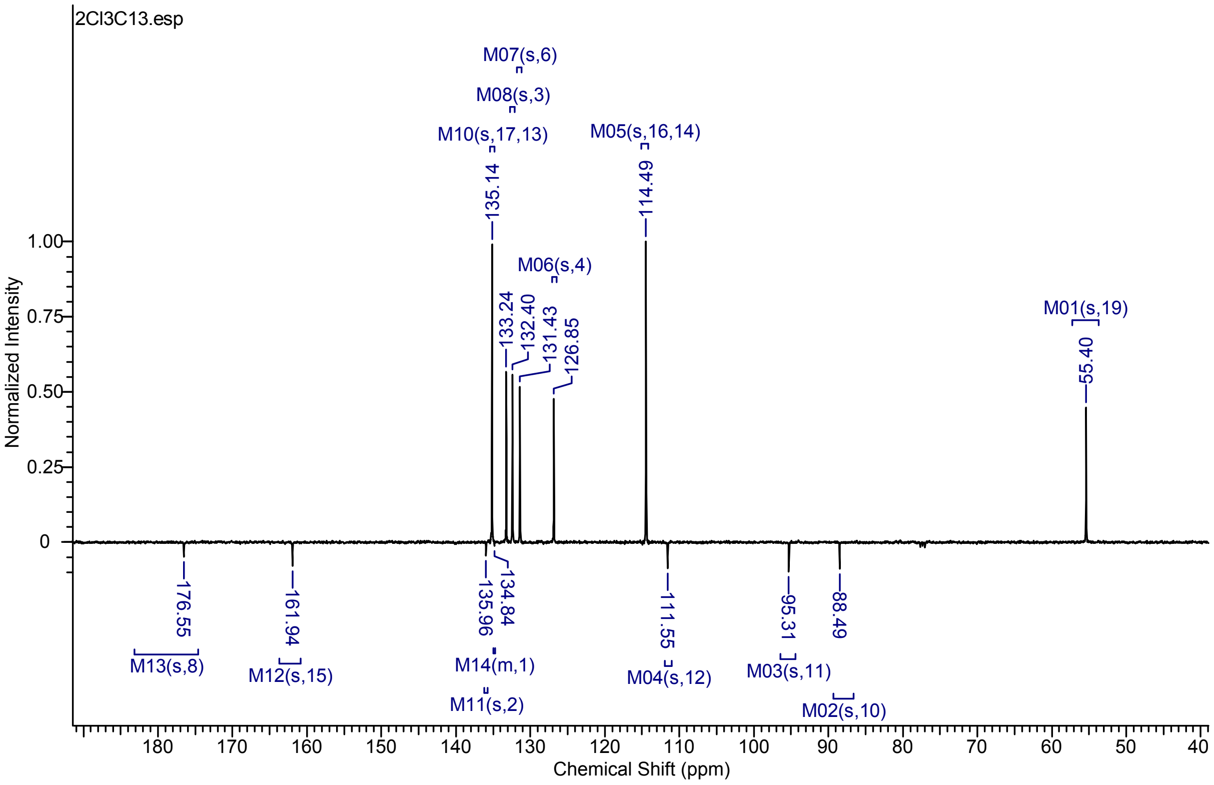

Figure S6. ^{13}C NMR analysis of compound **7**.

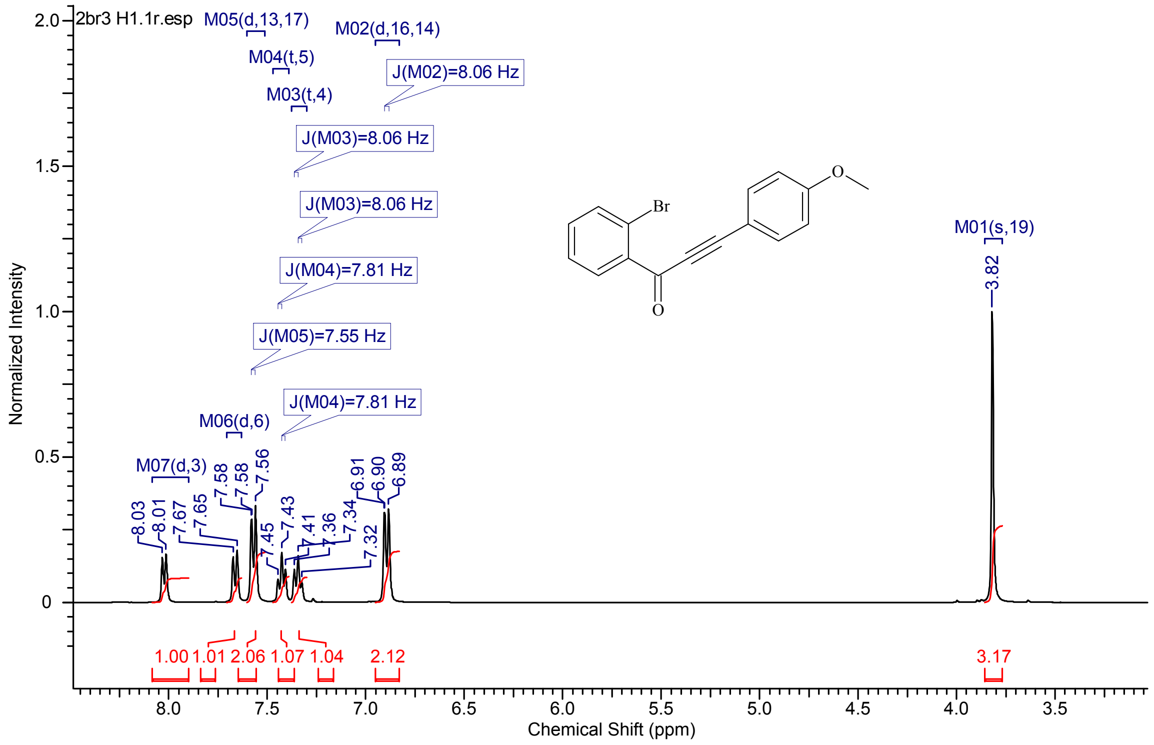

Figure S7. ^{1}H-NMR analysis of compound **8**.

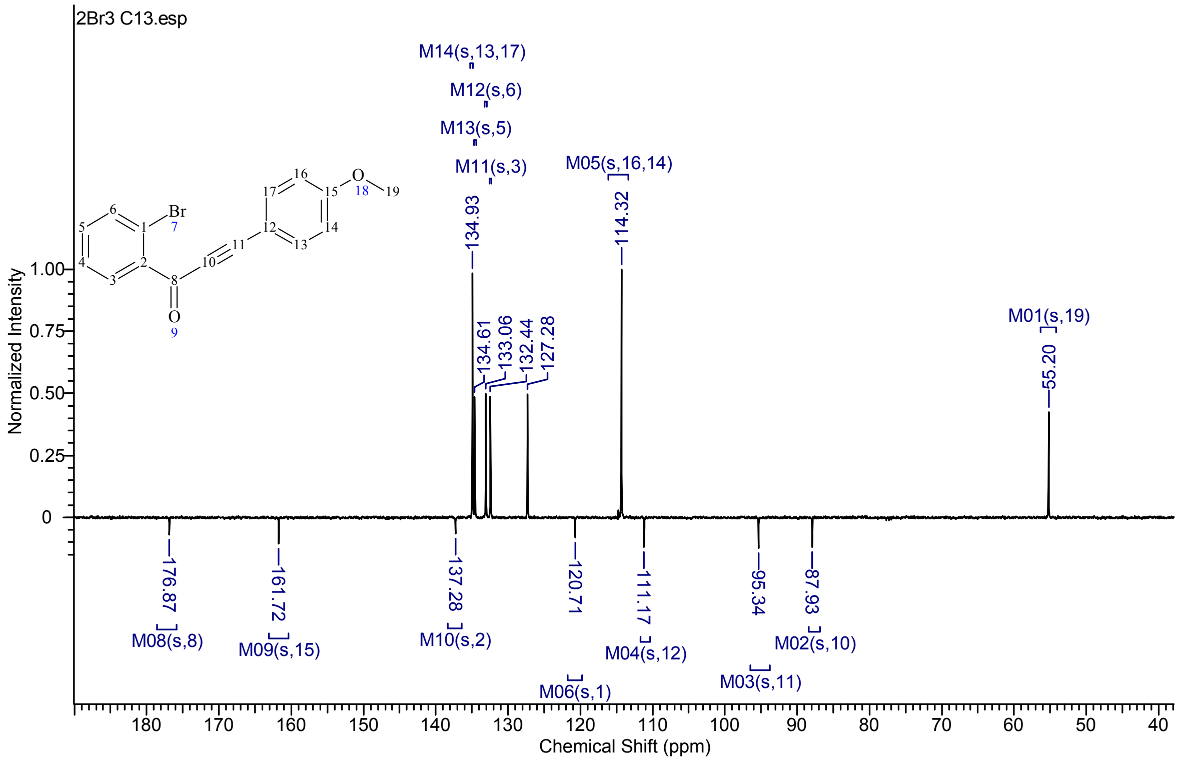

Figure S8. ^{13}C-NMR analysis of compound **8**.

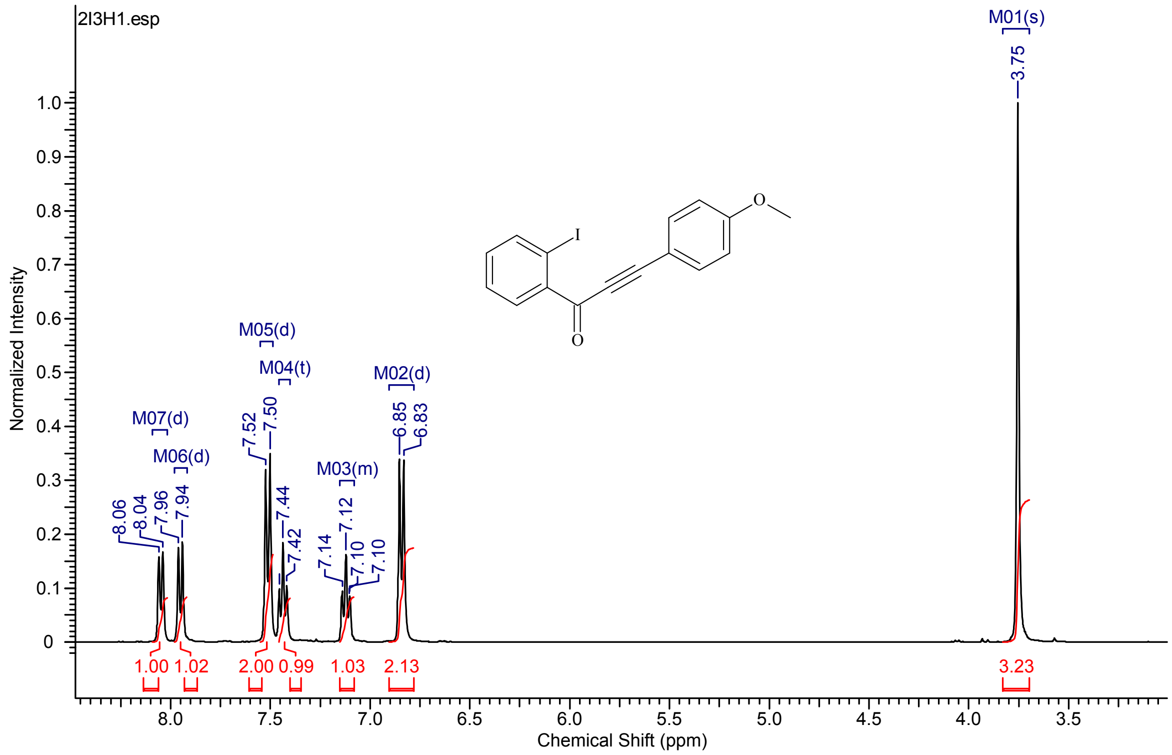

Figure S9. GC-MS analysis of compound **9**.

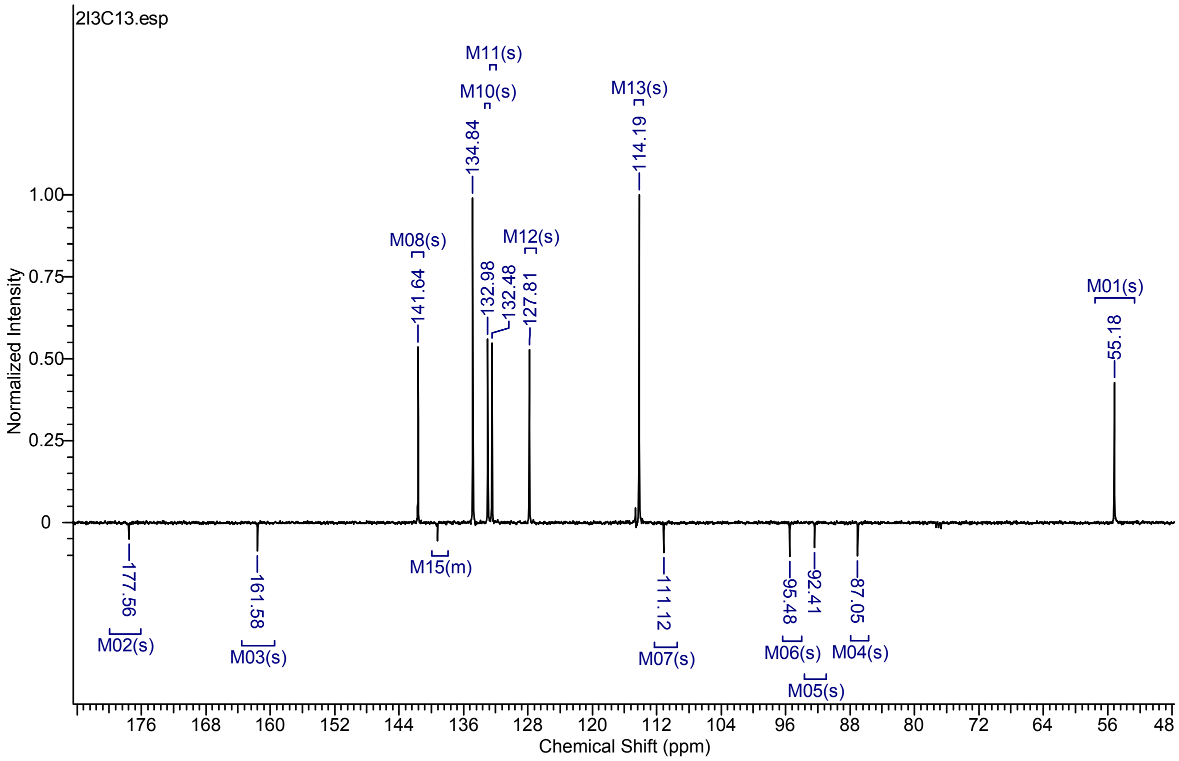

Figure S10. ^{13}C-NMR analysis of compound **9**.

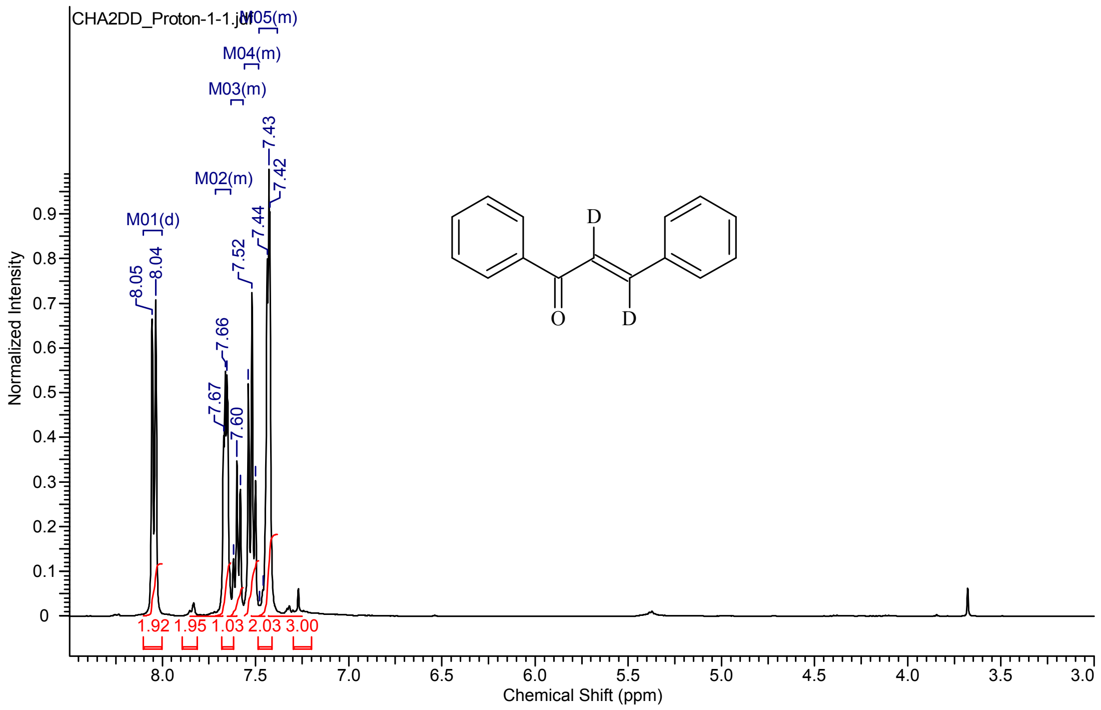

Figure S11. ^{1}H NMR analysis of compound **5a**.

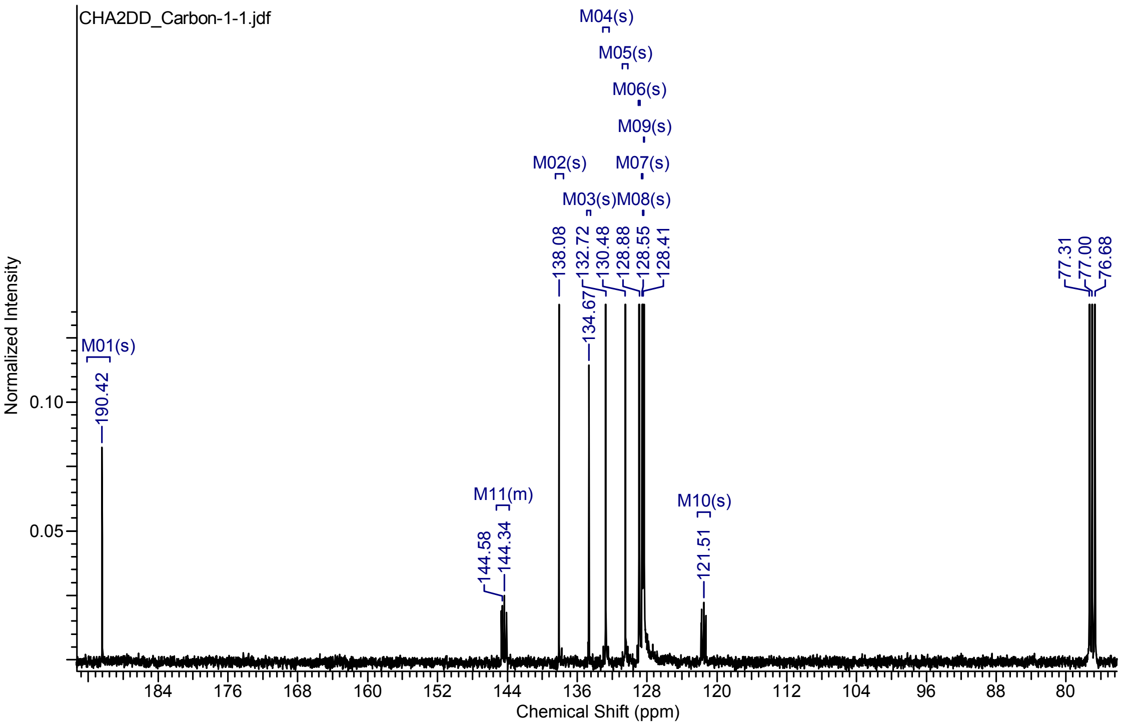

Figure S12. ^{13}C NMR analysis of compound **5a**.

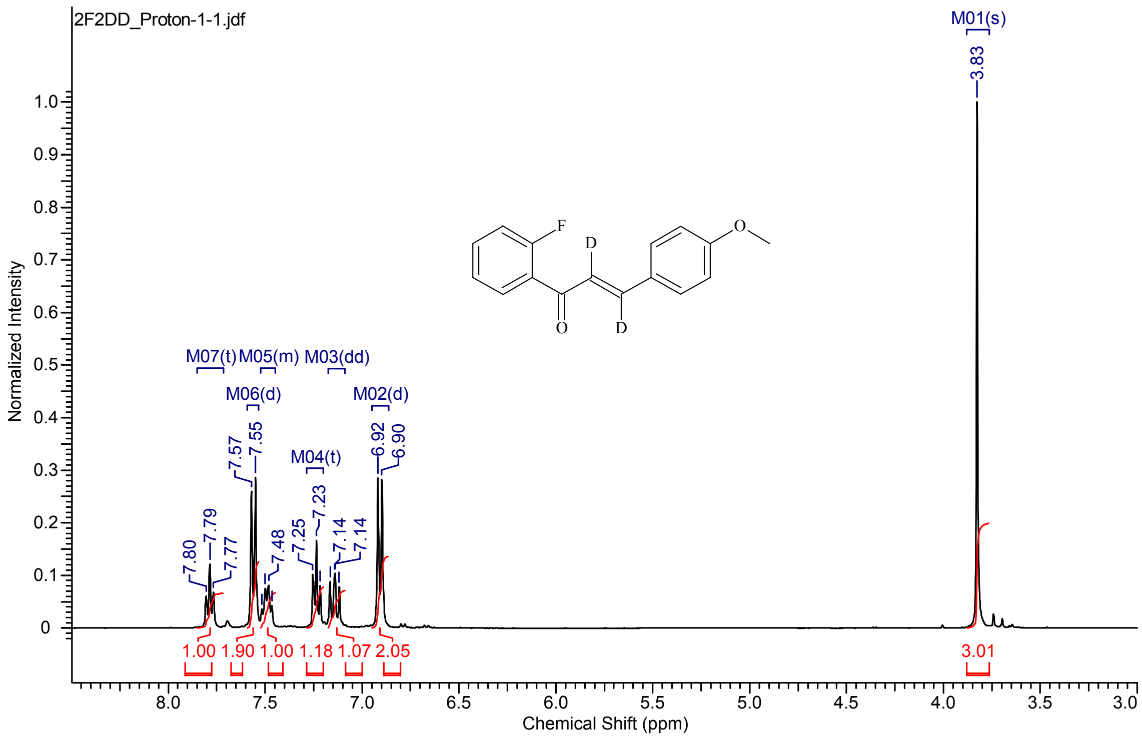

Figure S13. ^{1}H-NMR analysis of compound **6a**.

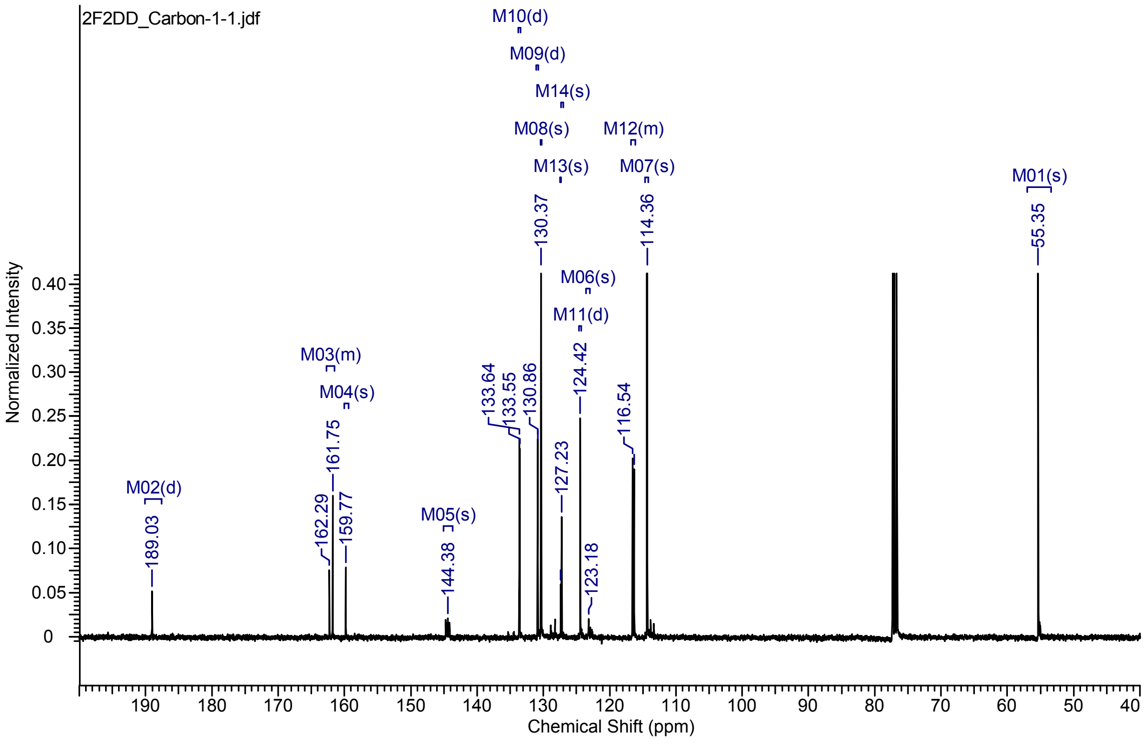

Figure S14. ^{13}C-NMR analysis of compound **6a**.

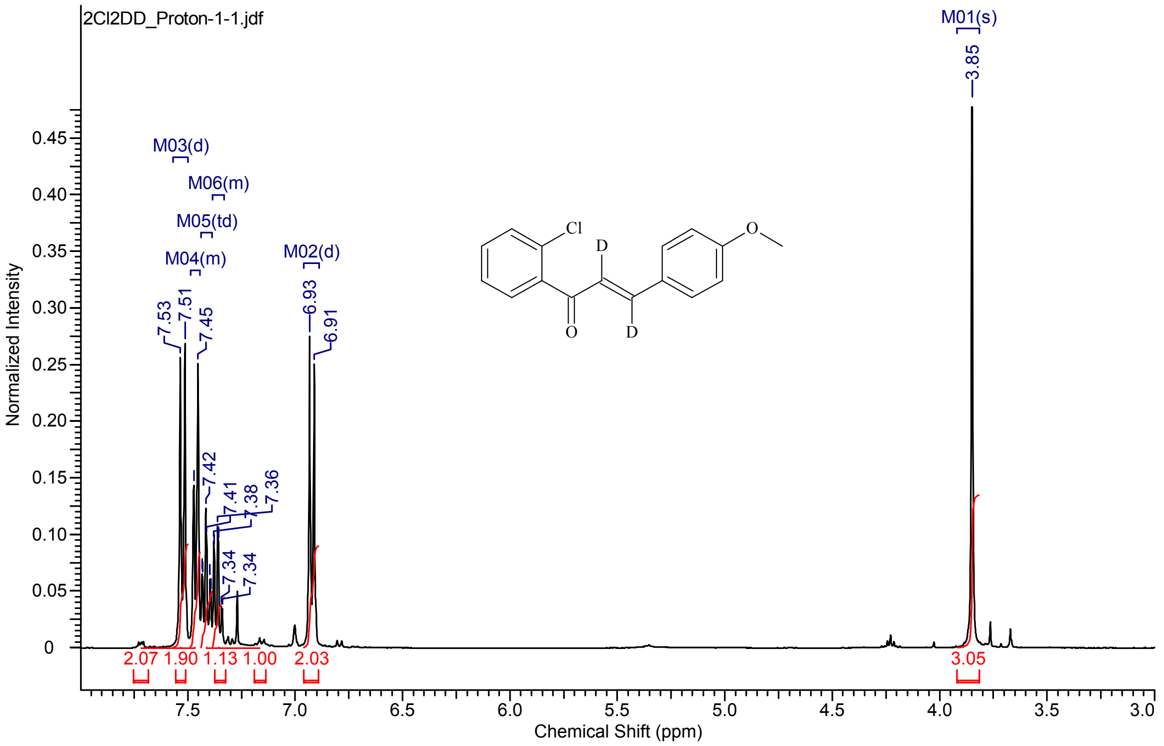

Figure S15. ^{1}H-NMR analysis of compound **7a**.

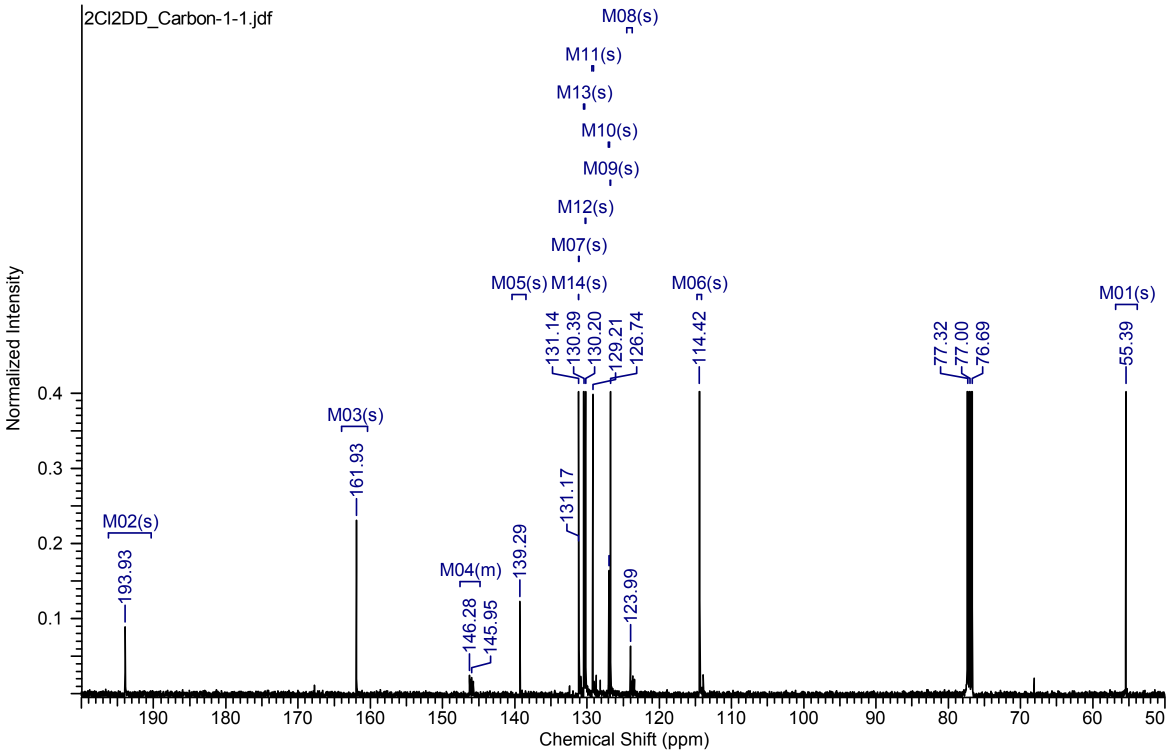

Figure S16. ^{13}C-NMR analysis of compound **7a**.

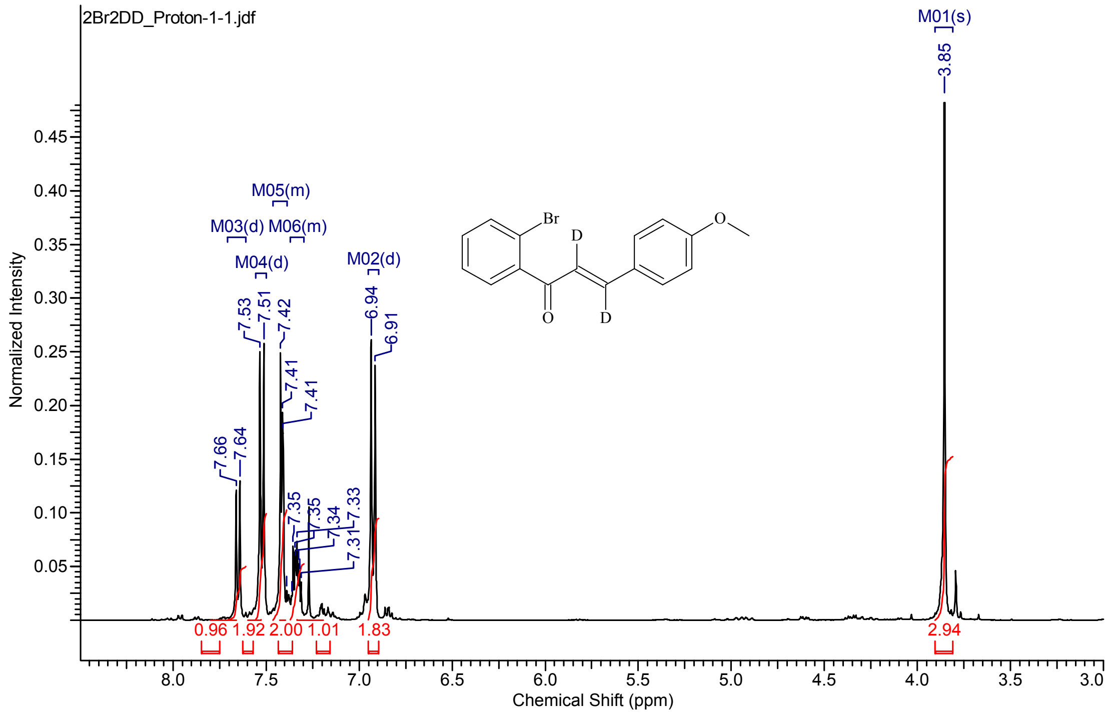

Figure S17. ^{1}H NMR analysis of compound **8a**.

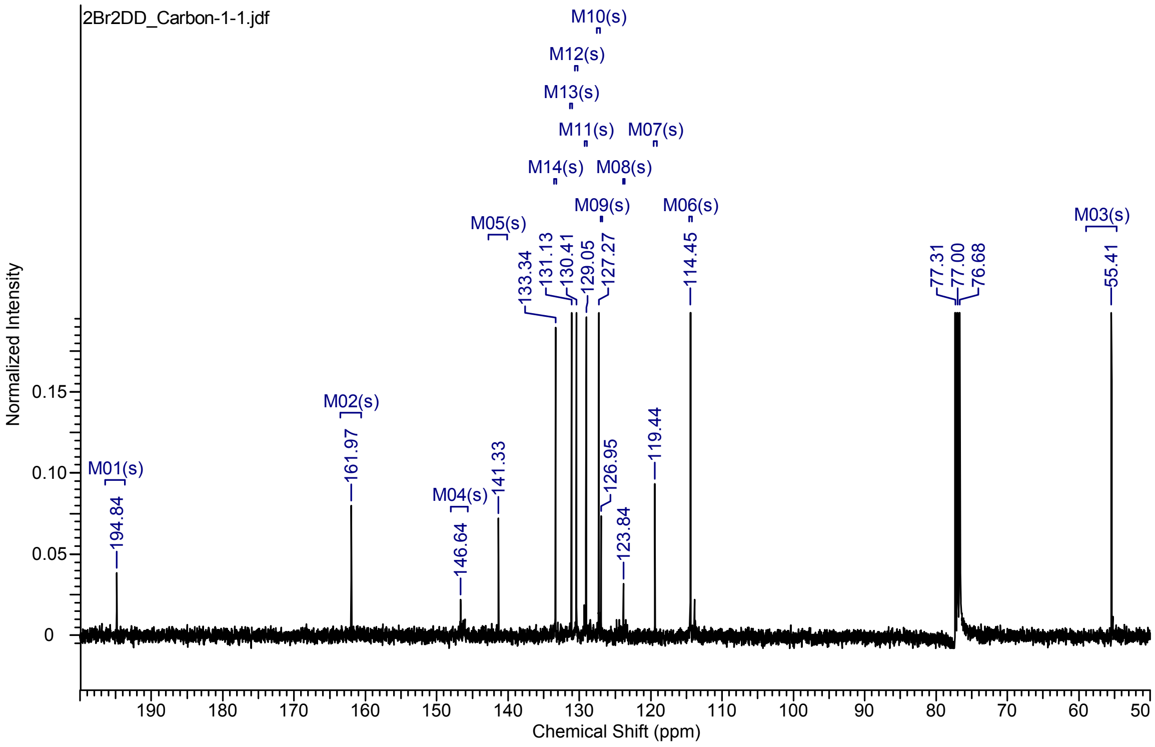

Figure S18. ^{13}C-NMR analysis of compound **8a**.

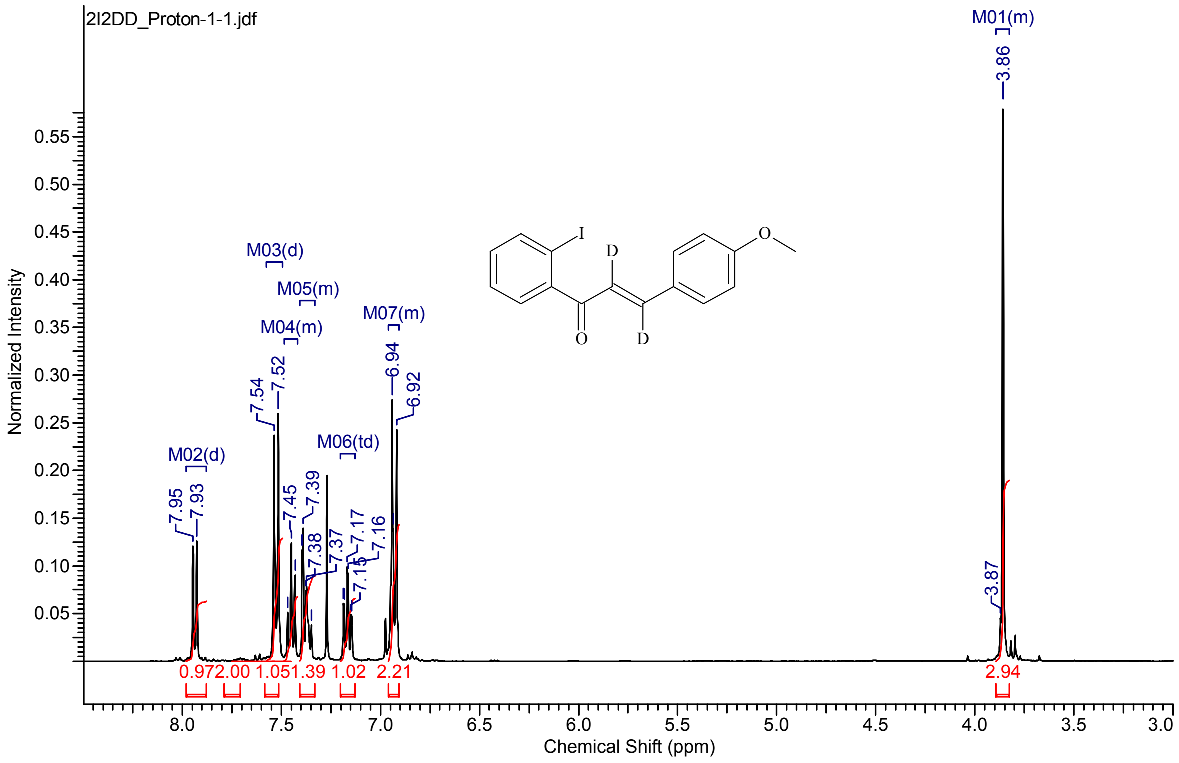

Figure S19. ^{1}H-NMR analysis of compound **9a**.

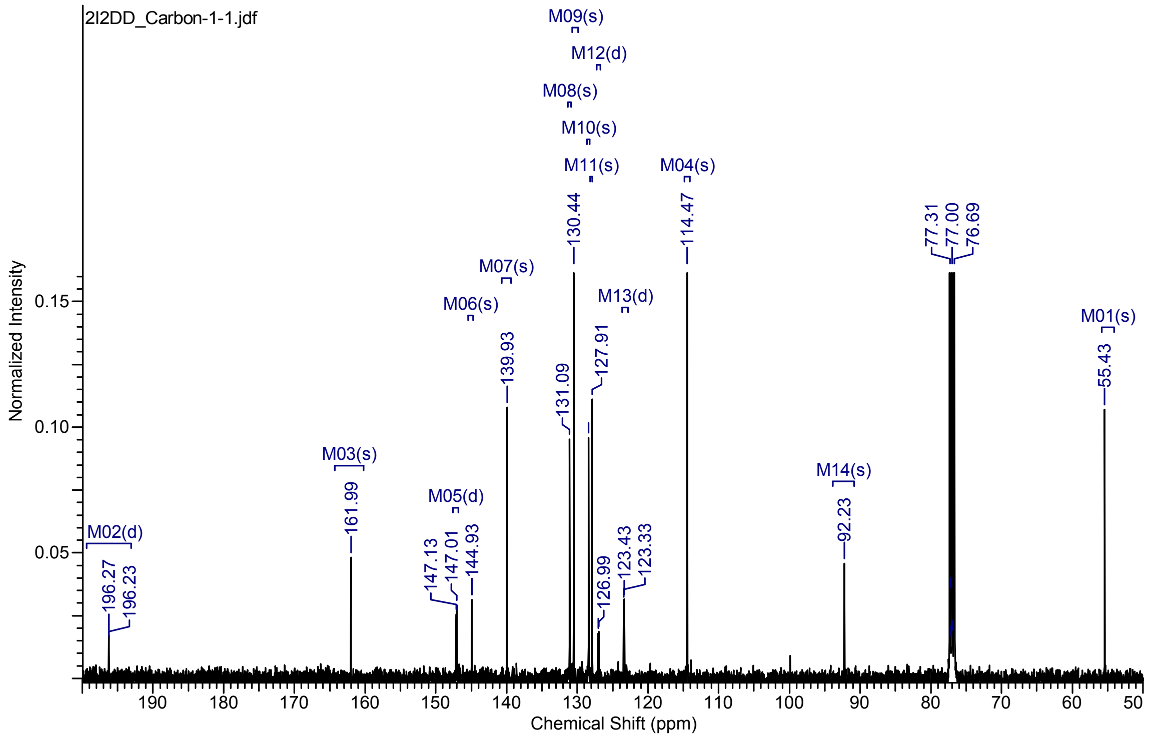

Figure S20. ^{13}C-NMR analysis of compound **9a**.

References

1. Chen, L.; Li, C.-J. A Remarkably Efficient Coupling of Acid Chlorides with Alkynes in Water. *Org. Lett.* **2004**, *6*, 3151–3153.
2. Shao, J.; Huang, X.; Hong, X.; Liu, B.; Xu, B. Synthesis of N-Alkyl-Substituted 4-Quinolones via Tandem Alkenyl and Aryl C–N Bond Formation. *Synthesis* **2012**, *44*, 1798–1805.
3. Chen, Y.; Huang, C.; Liu, X.; Perl, E.; Chen, Z.; Namgung, J.; Subramaniam, G.; Zhang, G.; Hersh, W. H. Synthesis of Dibenzocyclohepten-5-ones by Electrophilic Iodocyclization of 1-([1,1′-Biphenyl]-2-yl)alkynones. *J. Org. Chem.* **2014**, *79*, 3452–3464.
4. Cai, Q.; Zhou, F.; Xu, T.; Fu, L.; Ding, K. Copper-Catalyzed Tandem Reactions of 1-(2-Iodoary)-2-yn-1-ones with Isocyanides for the Synthesis of 4-Oxo-indeno[1,2-*b*]pyrroles. *Org. Lett.* **2011**, *13*, 340–343.
